# Supplementary material for: LC-MS/MS Analysis Elucidates a Daily Rhythm in Orexin A Concentration in the Rat Vitreous Body
Source: Molecules. 2021 Aug 19;26(16):5036. doi: 10.3390/molecules26165036 (PMC8401233; doi:10.3390/molecules26165036)
Supplement: Supplementary file 1 [file molecules-26-05036-s001.zip › molecules-1310971-supplementary.pdf]

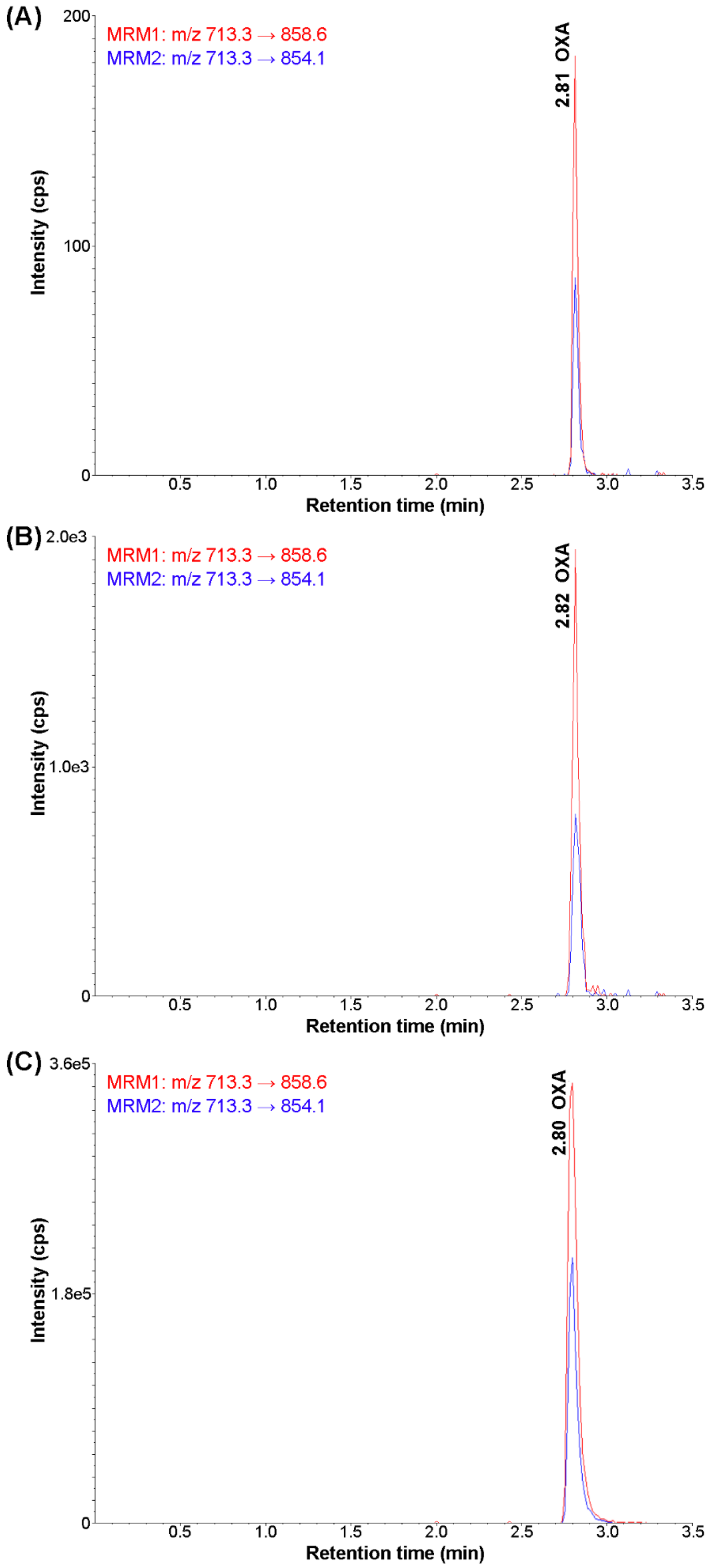

Figure S1. Representative MRM chromatograms of orexin A at different concentrations ((**A**) 10 pg/ml; (**B**) 100 pg/ml; (**C**) 400 pg/ml) (UHPLC-MS/MS).
